# The Unity of the Human Species
**Unité de l'Espèce Humaine.* Par A. de Quatrefages, Membre de l'Institut (Académie des Sciences). Paris, 1861.


**Published:** 1862-04

**Authors:** 


### Art. VI.—THE UNITY OF THE HUMAN SPECIES.*

It is an interesting illustration both of the importance of insignificant things, and of the gradual development and expansion of truths with which genius has toyed; that the unity of the human race, which, during the last century, was little more than a philosophical puzzle, is now a test of orthodoxy and a part of a political creed. It is more; for, in its practical bearings, the settlement of the question is the ostensible cause of what has been, in a more general and truthful significance than what was conceived by the name-giver, a fratricidal war; and the vast and vindictive hosts now on the eve of slaughter in America are nearly as well entitled to be arranged as monogenists and polygenists as the naturalists in M. Quatrefages' book. They offer wager of battle on the dicta that the black races are, or are not, men, brothers; that they are, or are not, cattle, chattels; sprung, or not, from the same stock as the white, the red, or the yellow races.

It was long ago whispered that the Adam and Eve of America were born in Newgate, and it would be as fair, according to our author, to adopt this sneer, as the colour of the skin as an argu-

---

* *Unité de l'Espèce Humaine.* Par A. de Quatrefages, Membre de l'Institut (Académie des Sciences). Paris, 1861.

ment for a distinct origin. M. Quatrefages, the distinguished contributor to the *Revue des Deux Mondes*, has produced, not a popular book, but a scientific treatise, which deserves to be most popular, in discussing from the philosophical point of view, the inquiries whether there was one common origin of mankind; whether all nations, tribes, peoples, however diversified in other respects, are different species or races, and descended from one primitive parentage or many. And it is worthy of consideration that he has been led by his researches, pursued by the aid of botany, zoology, history, &c., to the convictions which have the sanction of religion and of popular belief.

We discover, from the preliminary pages of the volume, that the speculation as to pre-Adamite man, which recently attracted attention, is originally due to La Peyrère, a protestant gentleman attached to the Prince of Condé. He attempted, in 1655, to show that the biblical narrative of Adam and his descendants is merely the commencement of the history of the Jews, and not of mankind. The previous creation was that of the Gentiles, contemporaneous with that of the animals on the sixth day, and belonging, as it were, to the genesis of nature. They were formed from the materials of chaos, had spread over the whole world, but had never penetrated into Paradise. The facts that the family of Adam was reduced to three persons by the death of Abel, yet that Cain was accompanied by his wife on his expulsion and flight, and that he was so marked by God as to be saved from the persecutions of his enemies, are held to show that other families must have co-existed, from which his wife and these persecutors could be drawn. Such is the class of arguments upon which this, the first polygenist, supports his theory. The controversy, however, which now rages, dates only from the moral and intellectual convulsion which accompanied the French Revolution, and was then what it has continued until recently to be, a mortal struggle between the theologians and the philosophers. The arena has changed not inappropriately to America; and M. Quatrefages openly charges the polygenists of that land, such as Nott, Gliddon, and Merton, with having been associated with, if not as being the direct agents of Calhoun, in advocating the perpetuation of slavery on the ground of the radical differences which separate the free and the fettered race.

While man possesses intelligence in common with animals, he is distinguished by those sentiments of morality and religiosity, by the recognition of something above him, which are found in lesser or greater activity in every individual of the race; but cannot be detected even in a rudimentary form in the most highly gifted brutes, even in the anthropoid apes, whose aspect has suggested the monkey basis of mankind. It appears, however, that although the emotions exist, there are no words signifying these qualities in the languages of Australia. Poverty in vocables explains this privation, which extends to common objects, such as a bird or a fish. The supposed atheistic populations have been vindicated. Campbell and Livingstone have shown that even the Hottentots and Caffres have a mythology; and that the absence of idolatry and public worship has been erroneously interpreted as a proof of disbelief in the supernatural.

The many-sided contest revolves upon a minute point; upon what constitutes a species, what a race. Is a species a group of individuals, more or less resembling each other, which have descended or may be regarded as descended from a single pair by an uninterrupted succession of families; and are races subordinate groups arranged according to the differences between the members, introduced and perpetuated by external circumstances?—according to Quatrefages; or, is a species a group of individuals characterized by identical and distinctive features, natural and transmissible, unaffected by external circumstances; so that wherever marked and permanent differences exist, as between the Caucasian and Australian, these must be accepted as indicating distinct species, originating in different parents—according to Lamarck, Isidore Geoffroy, and those who entertain polygenistic doctrines? It is the object of our author to demonstrate that the terms of the latter definition are untenable. He shows, and we conceive successfully, that the arguments in favour of the fixity and variableness of species are equipoised. The permanence of character appears to be established by the detection of the glumes of the barley, now cultivated, in the bread found in the Egyptian sarcophagi; measured by the layers of woody deposit in the trunk, the age of the yew at Foullebec may be 1200, of that at Fortingall, in Scotland, 3000; an *Adamsoria Baob* mounts to 5000; while the gigantic *Sequoia* of California is fresh and green, although its branches waved in the breeze 6000 years ago, in the time of the first Egyptian dynasties; but the seedlings and saplings which grow around resemble these veterans in every, the most minute, quality except size. The drawings in the tombs on the Nile show, in like manner, that the animals which they represent are precisely the same as those with which we are familiar. But, on the other hand, the variability of species finds support from such phenomena as that of melanism or the appearance of black-skinned individuals or families among white races, or of albinos among Africans; of the changes in the same individual in infancy, youth, manhood, under disease, &c.; the production of the elegant and universally cultivated shrub the *Robinia Spectabilis* without, from the *Robinia Pseudoacacia* with spines. M. Quatrefages, defining a variety as an individual or group of individuals proceeding from the same seminal origin, distinguished from the other members of the same species by exceptional characters, and a race as a number of similar individuals belonging to the same species, having received and transmitted by generation the characteristics of a primitive variety, proceeds to show the influence of the medium, or the circumstances, such as climate, government, social institutions, by which species are surrounded, in producing and developing such exceptional characteristics. He points to the ass, whose lineage is so certainly known, as an illustration of the results of physical agents, which is found ranging the west of Asia as the wild onagra, which is majestic in Persia, the size of a Newfoundland dog in the south of India, the depilated dwarf slave of our gipsies, and as shaggy as a goat in Poitou; he points to dogs, to the threadbare pigeon illustration, to the loss of hair by the ox, of feathers by the domestic fowl on being transported to meridional America; and then, indicating the part played by innate and hereditary tendencies in fixing and perpetuating at once a resemblance to and a difference from the parents, he enters upon the formation of animal races.

The actual creation of the Durham and Dishley breeds of cattle by selection or breeding in and in, or what the French more truly designate the "amelioration of the race through the family," may serve as an example of the class of arguments upon this point. The history of the porcupine, or what we would prefer to call the pachydermatous, and the many-fingered families; the increased stature in the population in and around Potsdam, from the experiments of the giant-breeding Frederick of Prussia; the bronzing of the skin, and crisping of the hair of travellers in Abyssinia; and many similar facts, are gathered into a proposition enunciating the deviations which may be effected within historic and brief periods, even under our own eyes, without affecting the integrity of the species, yet tending to establish distinctions.

It has been long known to ethnologists, that the pyramidal skull, the broad and large configuration of the jaws, zygomata, and face generally, compared with the size of the brain, of the nomadic Turkish tribes inhabiting Central Asia, give place in the long-civilized European Turk to the oval skull and features characteristic of the settled and educated races with which they are brought into contact. But the most pregnant and piquant illustration is that selected by M. Quatrefages from the organic changes going on in the community of which his chief opponents are members. The African arrives in America a true Negro. His first enslaved descendant presents softened lineaments; the face has ceased to be snout-like or muzzle-shaped; the head and body in each successive generation, approach the form of the European, and intellectually, and in despite of all physical and moral obstacles, the purest Creoles rise in the scale. But evidence is given that retributively the slave-owner falls, and approaches his bondman. The physical change consists in increase of stature and enlargement of the orbits; a diminution of the adipose tissue, and of the whole glandular system. "A few years have sufficed to establish a well-marked difference between the modern Americans and the English, of whom they are descendants; and apart from the European civilization which has followed them, we find some, after two centuries, with the facial angle the fierceness and the cunning of the Iroquois, others with the same exterior, the rudeness, the freedom, and independence of the Illinois and Cherokee," &c.*

We have not space to enter upon the many and interesting subjects involved in the investigation of the influence of the union of different races, of different species, or hybridization in animals; nor of the crossing of different varieties of men; not even of the theory of Agassiz, who pretends to hold that man may be of the same species, but that the blonde and the black, &c., have sprung from different stocks, in different regions, it may be in widely separated epochs, and that they have grown up and multiplied, their languages, as well as their habits and institutions, growing up with them—which have all an intimate connexion with the philosophy of mind.

That no attempt has been made to establish the identity of the races of man psychologically is extraordinary. M. Quatrefages approaches the subject,† but he cannot be said to have grappled with it, nor to have appreciated the powerful aid to the principle which he vindicates, that may be derived from its consideration. If the religious sentiment and the sense of responsibility separate man widely from the lower animals, the different members of the race, however greatly differing in colour, stature, even form, however modified by climate or other causes, are identified by possessing the same mental faculties, the same emotions, the same passions. This truth is so certain that it might be cast according to the formula of *Vigilantius, quod ubique semper et ab omnibus.* The earliest monuments, even when divested of sacred character and authority, the Vedas, the Zend Avesta equally with the Bible, when they speak of man, ay, and of whatever colour, describe him intellectually and morally the same as he is now: actually as well as potentially the same, for after making all due allow-

---

* M. l'Abbé Brasseur de Bourbourg: *Histoire des nations civilisées du Mexique et de l'Amerique centrale durant les siècles antérieurs à Christophe Colomb.*

† Pp. 158—169.

ance for the operation of the laws of descent acting through changes in organization, he is much less altered in intellectual and moral features than the grand original design appears to have contemplated. The neglect of this indication of unity is still more curious when it is considered that the works of the metaphysicians, historians, poets which analyse or paint man, of the most remote antiquity, though written by men of different races, or belonging to countries and climates remote from that centre towards which civilization has latterly gravitated, and, above all, uninfluenced by that marvellous religious and moral change which revolutionized Europe eighteen hundred years ago, enable us to form an adequate, even a microscopic estimate of the mental proportions of mankind. The capacity, the amount in which these powers are possessed and exercised will vary in different races and tribes, as they differ in different individuals of the same race, or tribe, or family, according to their physical condition and the amount of cultivation; but the nature, the influence of each, and the laws by which they are severally or conjointly regulated, are the same. The New Hollander may be a Bacon in embryo; at all events he feels pain, and joy, and grief as Bacon did; he remembers the past, he idealizes the future, he draws inferences, he protects his friend, or he betrays him, as Bacon may have done. The Charib who enumerated only the length of seven, did so by the same power and process which distinguish the most profound calculator. If the feeble love of parents and offspring did actually sully the institutions of Eastern half-reclaimed tribes, poverty and a low morality seem to have deadened these feelings among ourselves. Even our vices, our crimes, our mental diseases may be appealed to as establishing brotherhood. It is true that an enormous gap separates the civilized from the savage man, but it is more a historical than a psychological gap. There are periods in the progress of each people in which they morally resemble every other people. We have all been savages, lived in wigwams, scalped or been scalped; and the difference between what we are and what we were is that of growth, not of nature. The tendency of all ethnological inquiries appears to indicate an original unity of language; the exercise of the same power of suggesting vocal and written signs, harmonizes with this view. It is true that the American anthropologists, who hold the doctrine of centres of creation, and that varieties of mankind have sprung up in different regions, and with them a specific language, hold likewise that the growlings of the different species of bear, cat, &c., inhabiting all parts of the world, could be as easily and reasonably derived from each other, and by the same process as linguists establish relations between Greek and Sanscrit. But in employing such an argument, the distinction between natural and artificial language is altogether forgotten. The instinctive cry of the same species will be the same; even of different species it may be the same or assimilated, where the same feeling is expressed; but if the structure of all articulate language be essentially the same, and if such changes as may exist be the result of external circumstances, the nature of the mind whose operations are represented *must* have been the same.

---

